# Do reality distortions contribute to an increased risk of violent offending in schizophrenia? – a narrative review

**DOI:** 10.1192/bjo.2021.723

**Published:** 2021-06-18

**Authors:** Patrick McLaughlin

**Affiliations:** National Forensic Mental Health Services

## Abstract

**Aims:**

To critically examine the factors that drive an increased risk of violence in the schizophrenic population, with emphasis on the role played by reality distorted symptoms.

**Background:**

A multitude of studies have reported a positive association between schizophrenia and violence. Many of the risk factors for violence among the non-mentally disordered population, such as substance use, childhood conduct problems and victimisation, are the same as for persons with schizophrenia. There remains controversy however as to whether reality distorted symptoms themselves contribute to the increased risk of violence.

**Method:**

Relevant literature was identified through a search of the following databases: PubMed, EMBASE, and PsycINFO. Data were appraised and synthesised to provide a comprehensive overview of the current evidence base for the role of reality distorted symptoms in violence in schizophrenia.

**Result:**

Studies ascertaining the contribution of reality distorted symptoms in violent behaviour have produced contradictory results. At a population level, several epidemiological surveys have found little or no contribution for reality distorted symptoms. Such studies frequently show that violence can be accounted for almost entirely by other factors such as substance use and victimisation. However studies investigating relationships between clinical diagnoses and population-wide violence may be unable to detect association at the symptom level. A number of studies have found strong associations between schizophrenia and violence which was not explained by comorbid substance use and have shown strong associations between specific reality distorted symptoms (in particular persecutory delusions accompanied by anger) and violent behaviour.

**Conclusion:**

There is heterogeneity in the relationship between schizophrenia and violence. Factors that are associated with increased risk of violence among the schizophrenic population are also pertinent to those without mental disorders. With regards to the pathways to violence in schizophrenia the following conclusions may be drawn: there is an well-established increased risk of violence associated with schizophrenia which has been replicated in many studies; this risk is driven largely by substance use but other factors such as victimisation are also important; there is evidence that reality distorted symptoms, particularly persecutory symptoms, play a role in violent behaviour in some patients, particularly when co-occurring with anger; finally, there may be shared aetiological links between schizophrenia and antisocial behaviour.

